# Liuweizhiji Gegen-Sangshen beverage protects against alcoholic liver disease in mice through the gut microbiota mediated SCFAs/GPR43/GLP-1 pathway

**DOI:** 10.3389/fnut.2024.1495695

**Published:** 2024-12-13

**Authors:** Mingyun Tang, Long Zhao, Fuchun Huang, Tiangang Wang, Xu Wu, Shanshan Chen, Juan Fu, Chaoli Jiang, Shulin Wei, Xuseng Zeng, Xiaoling Zhang, Xin Zhou, Mei Wei, Zhi Li, Guohui Xiao

**Affiliations:** ^1^Department of Spleen and Stomach Diseases, The Affiliated Traditional Chinese Medicine Hospital, Southwest Medical University, Luzhou, Sichuan, China; ^2^The Key Laboratory of Integrated Traditional Chinese and Western Medicine for Prevention and Treatment of Digestive System Diseases of Luzhou City, The Affiliated Traditional Medicine Hospital, Southwest Medical University, Luzhou, Sichuan, China; ^3^Cell Therapy and Cell Drugs of Luzhou Key Laboratory, Department of Pharmacology, School of Pharmacy, Southwest Medical University, Luzhou, Sichuan, China; ^4^Department of Hepatobiliary Diseases, The Affiliated Traditional Chinese Medicine Hospital, Southwest Medical University, Luzhou, Sichuan, China; ^5^School of Integrated Traditional Chinese and Western Clinical Medicine, North Sichuan Medical College, Nanchong, Sichuan, China

**Keywords:** alcoholic liver disease, Liuweizhiji Gegen-Sangshen beverage, gut microbiota, SCFAs, GPR43, GLP-1

## Abstract

**Introduction:**

Alcoholic liver disease (ALD) is a pathological state of the liver caused by longterm alcohol consumption. Recent studies have shown that the modulation of the gut microbiota and its metabolic products, specifically the short-chain fatty acids (SCFAs), exert a critical role in the evolution and progression of ALD. The Liuweizhiji Gegen-Sangshen beverage (LGS), as a functional beverage in China, is derived from a traditional Chinese herbal formula and has been clinically applied for ALD treatment, demonstrating significant efficacy. However, the underlying mechanisms of LGS for alleviating ALD involving gut microbiota regulation remain unknown.

**Methods:**

In this study, an ALD murine model based on the National Institute on Alcohol Abuse and Alcoholism (NIAAA) method was established.

**Results:**

The results showed that oral LGS treatment dose-dependently alleviated alcoholinduced liver injury and inflammation in mice through decreasing levels of ALT, AST and proinflammatory cytokines (TNF-α, IL-6, IL-1β). LGS significantly improved liver steatosis, enhanced activities of alcohol metabolizing enzymes (ALDH and ADH), and reduced the CYP2E1 activity. Notably, regarding most detected indices, the effect of LGS (particularly at medium and high dose) was comparable to the positive drug MTDX. Moreover, LGS had a favorable effect on maintaining intestinal barrier function through reducing epithelial injury and increasing expression of occludin. 16S rRNA sequencing results showed that LGS remarkably modulated gut microbiota structure in ALD mice via recovering alcohol-induced microbial changes and specifically mediating enrichment of several bacterial genera (*Alloprevotella*, *Monoglobus*, *Erysipelatoclostridium Parasutterella, Harryflintia and unclassified_c_Clostridia*). Further study revealed that LGS increased production of SCFAs of hexanoic acid in cecum, promoted alcohol-mediated reduction of GRP43 expression in ileum, and increased serum GLP-1 level.

**Discussion:**

Overall, LGS exerts a remarkable protective effect on ALD mice through the gut microbiota mediated specific hexanoic acid production and GPR43/GLP-1 pathway.

## 1 Introduction

Excessive drinking is a global issue that has a severe impact on human health. The liver, as the main organ for metabolism and detoxification, is the primary target organ for alcohol damage. Alcoholic liver disease (ALD) is a spectrum of diseases that begins with alcoholic fatty liver (AFL) and may progress to alcoholic steatohepatitis (ASH), alcoholic liver fibrosis (ALF), alcoholic cirrhosis (AC), and in some cases, alcohol-related liver cancer (ARLC). Approximately 90 percent of individuals with excessive alcohol consumption manifest primarily with hepatic steatosis (1), and it is one of the leading causes of illness and death from liver disease worldwide preventably (2).

The pathogenesis of ALD is complex, which is tightly associated with alcohol and its metabolites mediated liver injury, and the inflammatory response to injury. In recent years, with the development of omics study, research based on the gut-liver axis theory has revealed the impact of changes in the gut microbiota on ALD, becoming an important driving force in exploring the pathogenesis of the disease. The gut microbiota and its metabolites can penetrate the intestinal epithelial barrier and enter the portal vein, linking the connection between the gut and the entire body. The balance of the gut microbiota is characterized by a high abundance of essential beneficial bacteria and a low abundance of pathogenic or conditionally pathogenic bacteria. And the disruption of this balance is closely related to the development and severity of various diseases (3). Current research has confirmed that the dysregulation of the gut microbiota is a key factor in the occurrence and development of ALD (4). Bacterial products, including the short-chain fatty acids (SCFAs) and so on, mediate host response and plays an important roles in ALD pathogenesis (2). Regulating gut microbiota and the gut-liver axis has emerged as a promising strategy for ALD treatment.

Liuweizhiji Gegen-Sangshen beverage (LGS), a nutraceutical beverage composed of six medicinal and edible plants (*Puerariae lobatae* radix, *Hoveniae* semen, *Imperatae* rhizoma, *Crataegi* fructus, *Mori* fructus and *Canarli* fructus), is derived from Traditional Chinese Medicine, and has demonstrated significant clinical therapeutic effects and minimal side effects in the handling alcoholic symptoms and ALD (5, 6). In previous studies, it has been demonstrated that in ALD rats, LGS exerted protective effects through attenuating oxidative stress, alleviating insulin resistance and lipid metabolism, and enhancing alcohol metabolism (7–10). The involved signaling pathways included AMPK/SREBP-1, P2 × 7R/NLRP3, PPARα, Ras/ERK and IRS-1/PI3K/AKT (7–10). In HO2 cells, LGS enhanced the alcohol metabolism through increasing the activity of ADH1 and ALDH2, and reduced CYP2E1-induced oxidative stress (11). However, whether LGS has an impact on the gut microbiota and gut-liver axis remains unexplored. In our previous study, it was found that LGS mainly contained flavonoids, polyphenols and polysaccharides (5, 12). The *in vitro* incubation of LGS polysaccharides has demonstrated good prebiotic activity, which drew our attention to its potential for regulating the gut microbiota (12). Therefore, although LGS has shown considerable clinical benefit toward ALD, the mechanism of action remains unclear and requires further elucidation.

In this study, we hypothesized that LGS may impact the gut microbiota, trigger host response and alleviate ALD. An ALD murine model was established by using Lieber-DeCarli alcohol liquid diet, and the mice were treated with different doses of LGS to investigate the effects of LGS on ALD. The results of current study would reveal the potential mechanisms of LGS in treating ALD from the aspect of gut microbiota regulation and provide a scientific basis for the clinical application and development of related drugs.

## 2 Materials and methods

### 2.1 Reagents and materials

LGS was provided by Sichuan Tongyou Life Health Technology Co., Ltd. (Sichuan, China). The chemical profile as well as quality control of LGS has been investigated thoroughly in our previous reports (5, 12). Metadoxine (MTDX) was purchased from MedChemExpress Co., Ltd. (New Jersey, US). Edible alcohol (95%) was acquired from Kelong Chemical Chemicals (Chengdu, China). Biochemical Assay Kits for TC, TG, ALT, AST, ADH and ALDH, Enzyme-linked immunosorbent assay (ELISA) kits for LDL-C, HDL-C, TNF-α, CYP2E1, and GLP-1 were provided by Quanzhou RuiXin Biotechnology Co., Ltd. (Fujian, China). 4% Paraformaldehyde from Sichuan Scientist Biotechnology Co., Ltd. (Sichuan, China). In histopathological examination and immunohistochemistry and immunofluorescence experiments, Hematoxylin (G1004) and Oil Red O stain (G1016) and Fixative were purchased from Wuhan Google Biotechnology Co., Ltd. (Wuhan, China). Primary Antibody, Occludin, ZO-1, and histological DAB Staining Kit purchased from Service Biotechnology Co., Ltd. (Wuhan, China). Secondary antibody, goat anti-rabbit IgG (Lianke Bio, GRA0072, 1:200, Hangzhou, China) were sourced from the respective suppliers. GPR43 polyclonal antibody (#19952-1-AP, 1:500) provided by Proteintech Group Co., Ltd. (Wuhan, China), and HRP conjugated affinipure goat anti-rabbit IgG (#BA1054) was provided by Boster Biological Technology Co., Ltd. (California, US). Anti-glyceraldehyde-3-phosphate dehydrogenase (GAPDH) antibody (AF2823) and Bicinchoninic acid (BCA) Protein Assay Kit was purchased from Beyotime Biotechnology Co., Ltd. (Shanghai China). The RIPA lysis buffer, protease inhibitor cocktail (100×), HRP-conjugated goat anti-mouse/Rabbit IgG (H + L) were sourced from Shanghai YaZi Bio-Pharmaceutical Technology Co., Ltd. (Shanghai China). Total RNA extraction kit was provided by TianGen Biochemical Technology Co, Ltd. (Beijing, China). The qRT-PCR kit was purchased from Vazyme Biotechnology Co., Ltd. (7E760L3, Nanjing, China). Primers were synthesized by Beijing Qingke Biotechnology Co., Ltd. (Beijing, China). FastPure Feces DNA Isolation Kit (YH-feces) was provided by Shanghai Major Yuhua Co., Ltd. (Shanghai, China). ECL luminescence detection kit was purchased from Affinity Biotech Co., Ltd. (Jiangsu, China).

### 2.2 Animals and experimental design

A total of 72 male C57BL/6J mice (specific pathogen free grade) aged between 6 to 8 weeks, with a body weight exceeding 20 grams, were provided by the Animal Center of Southwest Medical University (Sichuan, China). The experimental protocol was complied with the guidelines for the care and use of laboratory animals set forth by the National Institutes of Health, and has been rigorously reviewed and approved by the Experimental Animal Ethics Committee of Southwest Medical University, with the corresponding approval number: SWMU20230089. All mice were maintained at the Animal Center of Southwest Medical University, with a temperature of 23 ± 1°C, a humidity of 40–60%, and 12/12 h day/night turnover. The mice were subjected for adaptation for at least 1 week before experiment with free access to water and food.

**FIGURE 1:**
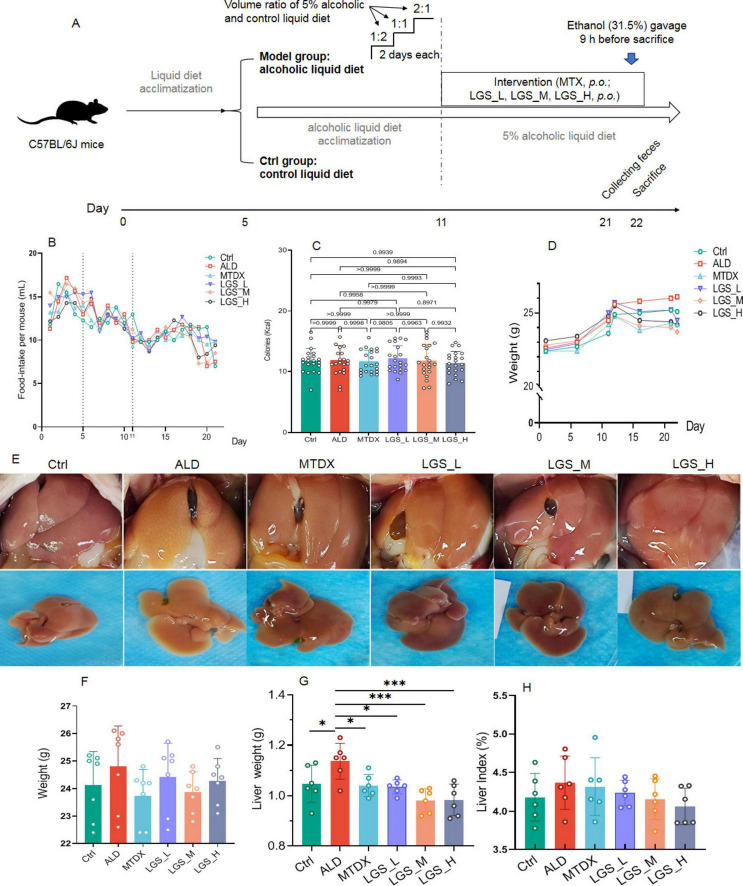
LGS ameliorates body and liver weight gain, and improves liver morphology in ALD mice. **(A)** Experimental procedure for animal studies. **(B)** Daily food intake of mice in each group. **(C)** Calories intake in each group. **(D)** The effect of LGS on the body weight of mice with ALD. **(E)** Trend of body weight change in each group. **(F)** Morphological presentation of liver images in each group. **(G)** Liver weight of mice. **(H)** Hepatic index (the ratio of liver weight to body weight) of mice. *P < 0.05, ***P < 0.001 (*n* = 6). One-way ANOVA followed by Tukey test was employed to evaluate differences among multiple groups.

Following the recommendations of the National Institute on Alcohol Abuse and Alcoholism (NIAAA) (13), we established a murine model of ALD (Figure 1A) using the Lieber-DeCarli alcoholic liquid diet (#TP4030D; Trophic Animal Feed High-Tech Co., Ltd., Jiangsu, China). The complete alcoholic liquid diet was prepared with food-grade 95% ethanol, with a final alcohol concentration of 5% (v/v), which was consisted of 28% calories from alcohol, 35% calories from fat, 18% calories from protein and 19% calories from alcohol carbohydrate. In the control liquid diet (#TP4020C; Trophic Animal Feed High-Tech Co., Ltd., Jiangsu, China), equal calories for alcohol were replaced by dextrin. All liquid diets were freshly prepared daily at a caloric density of 1 kcal/mL.

In the initial phase of the study, all mice were acclimated to the experimental environment for 1 week, followed by a 5-day period (days 1–5) of adaptation to liquid diet feeding. Then the mice were randomly assigned into six groups (*n* = 12 per group), comprising a control group (Ctrl), an ALD model group (ALD), a MTDX treatment group (MTDX), and three LGS treatment groups (LGS_L, LGS_M, LGS_H). The sample size was estimated by the free G*Power software (Universität Düsseldorf), with effect size setting at 0.25, α probability of 0.05 and power of 0.95.

From days 6 to 11, except for the Ctrl group which received control liquid diet, the remaining five groups were subjected to a graded transition feeding with the mixture of control liquid diet and alcoholic diet at the volume ratios of 2:1, 1:1, and 1:2 (each for 2 days). The Ctrl group was pair-fed according to the mean food intake of the ALD groups from the previous day, in order to ensure similar food intake for Ctrl and ALD mice. During days 12 to 21, the ALD, MTDX and LGS treatment groups were administered the complete alcoholic liquid diet, while the Ctrl group continued with pair-feeding for a total of 10 days.

Throughout this period, the Ctrl and ALD groups were daily gavage-fed with physiological saline, the MTDX group with metadoxine solution (200 mg/kg/day), and the LGS_L, LGS_M, and LGS_H groups with LGS at low, medium and high dosages of [6.15 mL (1.23 g solid content)/kg/day, 12.30 mL (2.46 g solid content)/kg/day, 24.60 mL (4.92 g solid content)/kg/day], with a gavage volume of 0.2 mL per mouse per day. The dosage of LGS used in mice was designed according to the human dose and cross-species dose conversion using body surface area scaling. Consequently, the dosages for LGS_L, LGS_M, and LGS_H corresponded to 0.5-fold, 1-fold and 2-fold of normal human daily dose. On the 21st day of the experiment, fresh fecal pellets from the mice were collected and stored at −80°C. On the 22nd day, the mice were euthanized for blood collection. After sacrifice of mice, liver and intestine tissues, as well as intestinal contents were collected for subsequent analysis. During the course of the study, each animal was weighed at 5-day intervals.

### 2.3 Biochemical analysis

Blood samples were centrifuged at 4°C, 4000 rpm for 20 min to collect the serum. Serum levels of liver function-related indicators (AST and ALT), blood lipids (TC, TG, LDL-C and HDL-C), proinflammatory cytokines (IL-6, IL-1β and TNF-α), and alcohol metabolizing enzymes (CYP2E1, ADH, ALDH and GLP-1) concentrations were determined using the manufacturer’s protocol with the respective ELISA or biochemical assay kits. The optical density (OD) of the plates was read at 450 nm for ELISA kits and 462 nm for biochemical assay kits, using a microplate reader, and the concentrations were calculated by standard curves.

### 2.4 Histopathological examination

The liver and intestinal tissues were fixed overnight in 4% paraformaldehyde and then embedded in paraffin. Sections of the liver and ileum were stained with hematoxylin and eosin (HE). To assess hepatic lipid accumulation, liver samples were frozen in optimal cutting temperature (OCT) compound and sectioned using a cryostat. After air-drying, the sections were fixed and stained with oil red O (ORO) in propylene glycol, followed by counterstaining with hematoxylin to highlight lipid deposition and cell nuclei. Microscopic images were captured, and the area ratio of positive expression within the tissue was measured using Image-Pro Plus 6 software (Media Cybernetics, Inc, Rockville, MD, USA).

### 2.5 Immunohistochemical and immunofluorescence staining

Immunofluorescence staining was performed on colonic sections to detect tight junction proteins (Occludin and ZO-1). The tissue sections were incubated overnight with primary antibodies specific for Occludin (1:100) and ZO-1 (1:100) at 4°C. Subsequently, the sections were incubated with a secondary antibody, goat anti-rabbit IgG (GRA0072, 1:200), followed by a chromogenic reaction with 3,3′-diaminobenzidine (DAB, DA1016) for 45 min. Microscopic images were captured and analyzed using a digital pathology system (3DHIST ECH Kft Pannoramic SCAN II).

### 2.6 RNA isolation and real-time PCR analysis

RNA was isolated from liver and intestinal tissues using the total RNA Extraction Kit. Quantitative PCR (qPCR) was performed using a Roche fluorescence quantitative system (Life Science and Technology, Veriti 96-Well) to assess the mRNA expression of the gene encoding GPR43. Primer sequences were as follows: *GAPDH*, 5′-ACTGAGCAAGAGAGGCCCTA-3′ and 5′-CCCTAGGCCCCTCCTGTTAT-3′; *GPR43*, 5′-ATCC AACTTCCGCTGGTACC-3′ and 5′-GTAGCGTTCCATG CTGATGC-3′.

### 2.7 Western blot

According to the manufacturer’s instructions, the ileal tissue (50 mg) was homogenized with RIPA lysis buffer and a 100 × protease inhibitor cocktail (v/v = 1:1) to obtain the ileal tissue lysate. Protein concentration was measured using the BCA Protein Assay Kit.

An equal amount of protein was separated by SDS-PAGE, and western blot analysis was performed using the specific primary antibody, GPR43 (1:1000), GAPDH (1:2000), and a horseradish peroxidase (HRP)-conjugated secondary antibody (rabbit pAb, 1:5000). The protein levels were quantified by densitometry scanning.

### 2.8 Gas chromatography-mass spectrometry (GC–MS) analysis

After re-suspending the cecal contents in a phosphate buffer, the supernatant was taken for analysis. Separation was performed using an Agilent DB-FFAP capillary column (30 m × 250 μm × 0.25 μm) and an Agilent 789B GC System. Nitrogen with flow at 1.0 mL/min as the carrier gas (Initial temperature was 90°C, raised to 160°C at 10°C /min, then raised to 240°C at 40°C /min, and held for 5 min). Subsequent mass spectrometry was conducted using an Agilent 5977B GC/MSD mass spectrometer (SCAN/SIM, inlet temperature 250°C, ion source temperature 230°C, transfer line temperature 250°C, quadrupole temperature 150°C, electron impact ionization (EI) source, electron energy 7 eV). The concentrations of SCFAs in the cecal contents were reported in milligrams per gram of feces (μg/g) by ChemStation software (Agilent Technologies, USA) on APT Cloud Platform.^1^

### 2.9 16S rRNA gene sequencing analysis

Total bacterial DNA was extracted using the FastPure Feces DNA Isolation Kit (YH-feces, Shanghai Major Yuhua). The quality of DNA extraction was verified by 1% agarose gel electrophoresis, and the quantity was determined using the QuantiFluor™ -ST Blue Fluorescence Quantitative System (Promega Corporation). Library preparation and sequencing were conducted at Majorbio Bio-pharm Technology Co. Ltd. (Shanghai, China). PCR amplification of the V3-V4 region was performed using the primers 338F (5′-ACTCCTACGGGAGGCAGCAG-3′) and 806R (5′-GGACTACHVGGGTWTCTAAT-3′) to prepare amplicons. The libraries were ultimately submitted for sequencing on the Illumina NextSeq 2000 PE300 platform (Illumina, San Diego, USA).

The paired-end (PE) reads obtained from Illumina sequencing were demultiplexed and subjected to quality control and filtering based on sequencing quality. The reads were then assembled based on the overlap between the paired-end reads to yield optimized data. Subsequently, the optimized data were processed using the sequence denoising method (DADA2) (14) to obtain amplicon sequence variants (ASVs) and their abundance information.

The raw paired-end reads from the Illumina platform were overlapped and merged using FLASH (v1.2.11) (15) with standard parameters. The merged reads were subjected to quality control using the QIIME2 platform. To minimize the impact of sequencing depth on subsequent alpha and beta diversity data analysis, all sample sequences were rarefied, and all data analyses were performed on the Majorbio Cloud Platform^2^ based on the Silva 16S rRNA gene database (version 138). The ASVs were classified taxonomically using the Naive Bayes classifier in Qiime2. The composition of the microbial community, alpha diversity indices (Mothur v1.30.2) (16), and beta diversity (Bray-curtis) indices were calculated using QIIME2. The similarity of microbial communities between groups was determined by principal coordinate analysis (PCoA) (Vegan v2.4.3 package). The linear discriminant analysis (LDA) effect size (LEfSe)^3^ was performed to identify the significantly abundant taxa (phylum to genera) of bacteria among the different groups (LDA score = 3.5, *P* < 0.05).

### 2.10 Statistical analysis

Statistical analysis was performed using GraphPad Prism version 8 (GraphPad Software Inc., USA) and SPSS version 20 (SPSS, USA). One-way analysis of variance (ANOVA) was employed to evaluate differences among multiple groups, and the unpaired student’s *t*-test was used to assess the statistical significance between two groups. The results are presented as the mean ± the standard error of the mean (SD). Data were considered statistically significant when the *p*-value was less than 0.05.

## 3 Results

### 3.1 LGS protects against ALD in mice

In this study, we administered the LGS to a murine model of ALD for a 10-day treatment period to investigate the therapeutic effects of LGS on ALD. The experimental results are depicted in Figure 1. Throughout the study, body weight of mice was monitored. The liver morphology was observed, and liver indices were determined. Subsequently, the hepatic histopathological analysis, biochemical determinations, and ethanol metabolism assays were performed.

#### 3.1.1 LGS ameliorates hepatic steatosis in ALD mice

In the initial 11 days of the experiment, corresponding to the liquid transition and alcohol acclimation period, there was a certain degree of fluctuation in the food intake of all groups. Despite a downward trend observed on the 5th–20th day, may be related to the alcohol intake, the food intake became more stable in general and with no difference between groups (Figure 1B). Moreover, to ensure that each group had similar calories intake, the amount of liquid diet supplied to the control group was given based on the amount of food consumed by the ALD group the day before. The results showed that there was no statistical difference in calories between the groups (Figure 1C).

As extrinsic indicator of obesity, the body weight of all groups showed an upward trend (Figure 1D), compared with Ctrl group, the ALD group having a higher average body weight. After intervention with LGS, the body weight of mice became lower. The MTDX group exhibited a loss of weight too (Figure 1F). Subsequently, we observed the *in vivo* and *ex vivo* images of the liver (Figure 1E). The livers of Ctrl and the medium and high dose LGS treatment groups (LGS_M, LGS_H) appeared red with sharp edges, in contrast, the livers of the ALD group were yellow, with blunt edges and a greasy texture. The MTDX group exhibited slight yellow areas with sharp edges. The livers of the LGS_L group were pale yellow with a few yellow areas, and the edges were slightly blunt. Further analysis revealed that, the ALD group mice had a larger liver weight (*p* < 0.05) (Figure 1F) and liver index (Figure 1G). After the treatment with LGS, the liver weight was reversed, with the liver index decreased. The improvement in the liver index showed a dose-dependent trend. The aforementioned findings indicate that LGS intervention apparently ameliorates body and liver weight gain, and improves liver morphology.

**FIGURE 2:**
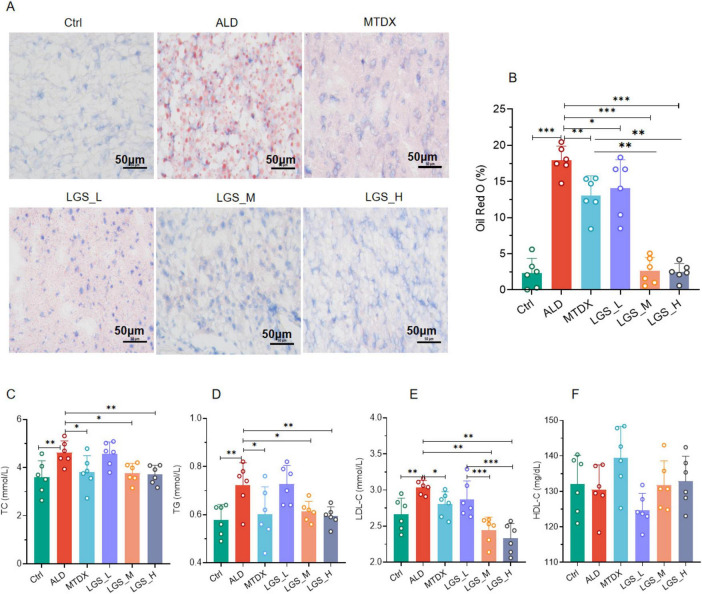
LGS ameliorates hepatic steatosis in ALD mice. **(A)** The effect of LGS on the Oil Red O staining of the livers of mice with ALD. **(B)** The proportion of the positive area of Oil Red O staining in the livers of mice in each group. **(C)** Serum TC of mice. **(D)** Serum TG of mice. **(E)** Serum LDL-C of mice. **(F)** Serum HDL-C of mice (*n* = 6, *P < 0.05, **P < 0.01, ***P < 0.001). One-way ANOVA followed by Tukey test was employed to evaluate differences among multiple groups.

Furthermore, liver tissues were sectioned and stained with Oil Red O (Figure 2A). The results revealed that a significant aggregation of red lipid droplets was observed in the liver of ALD group mice. A moderate aggregation was observed in the liver of LGS_L group mice. A slight aggregation was noted in the liver of MTDX group mice, and minimal aggregation was seen in the liver of Ctrl group, LGS_M group, and LGS_H group mice. To demonstrate the fat content within tissues directly, the ratio of the area of positive Oil Red staining to the entire observed area (positive expression area ratio,%) was analyzed. It was found that, in comparison to the Ctrl group, the fat content in the ALD group elevated significantly, and the positive area ratio reduced after LGS treatment (Figure 2B). Noteworthy, the effect of LGS was markedly superior to that of MTDX, particularly in the medium and high-dose groups.

Further confirmation was provided by serum biochemical assays, showed that the lipid levels of TC (Figure 2C), TG (Figure 2D), and LDL-C (Figure 2E) in ALD group mice were significantly elevated compared to the normal group (*p* < 0.05). Blood lipids were reduced by both medium and high doses of LGS and MTDX (*p* < 0.05). However, the experiment did not reveal the alleviating effects of LGS and MTDX on HDL-C (Figure 2F).

These above findings indicate that LGS is capable of ameliorating hepatic steatosis in mice with ALD, and, in blood lipid reduction, the data endorsed the use of higher dosages more.

#### 3.1.2 LGS protects against liver injury and inflammation in ALD mice

**FIGURE 3:**
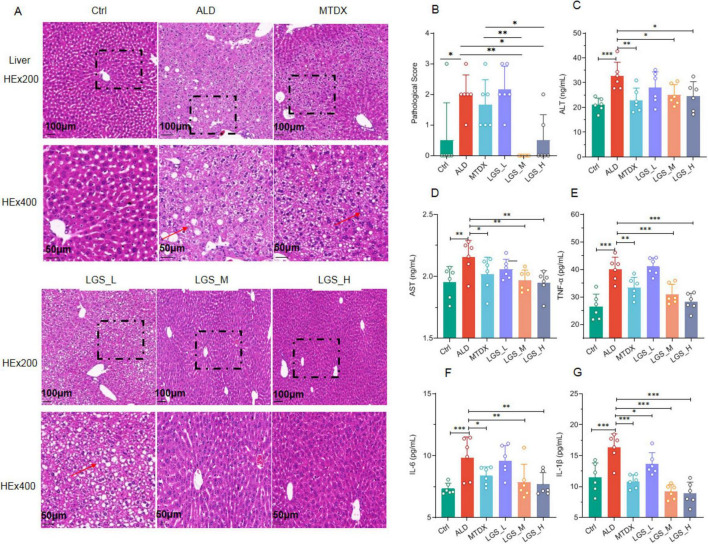
LGS ameliorates liver damage and inflammation in ALD mice. **(A)** Hematoxylin and Eosin (H&E) staining of mouse liver tissues at original magnifications of 200× and 400×. **(B)** Pathological scoring of H&E staining. **(C)** Serum ALT. **(D)** Serum AST. **(E)** Serum TNF-α in ALD mice. **(F)** Serum IL-6. **(G)** Serum IL-1β (*n* = 6, *P < 0.05, **P < 0.01, ***P < 0.001). One-way ANOVA followed by Tukey test was employed to evaluate differences among multiple groups.

Hepatic histopathological analysis and biochemical assays of blood samples were subsequently conducted to evaluate the hepatoprotection of LGS (Figure 3).

Compared to the Ctrl group, the ALD group exhibited a disordered arrangement of hepatocytes, significant cell swelling, and scattered vacuole formation, which indicated the presence of liver inflammation. This suggested that the inflammatory damage was present in ALD, which confirmed by pathological scores among groups. After intervention with LGS or MTDX, the hepatocyte arrangement became tightly and orderly, the structure of the liver lobules was clear, the phenomenon of cell swelling was not obvious, and the number of vacuoles was correspondingly reduced (Figure 3A). Furthermore, the effect of medium and high doses of LGS was significantly superior to that of MTDX regarding the improvement in liver injury (Figure 3B) (*p* < 0.05).

Two enzymes, ALT and AST, are predominantly located within cells, especially the hepatocytes. Upon liver cell damage, those enzymes are released into the blood, resulting in abnormally elevated levels of serum ALT and AST, which are frequently utilized as clinical biomarkers for liver injury. The results demonstrated that the serum levels of ALT (Figure 3C) and AST (Figure 3D) were elevated significantly in ALD mice in contrast to the Ctrl group (*p* < 0.05). Inflammatory responses are usually accompanied by the production of inflammatory mediators. We further measured certain inflammatory cytokines in the serum, and the results showed that the levels of TNF-α (Figure 3E), IL-6 (Figure 3F), and IL-1β (Figure 3G) were increased (*p* < 0.05) in ALD mice. Notably, after treatment with LGS or MTDX, these serum indicators were all significantly improved, with LGS_M and LGS_H groups sowing the best efficacy.

Overall, LGS exerts a protective effect on liver injury and inflammation in ALD mice.

#### 3.1.3 LGS enhances alcohol metabolism in ALD mice

**FIGURE 4:**
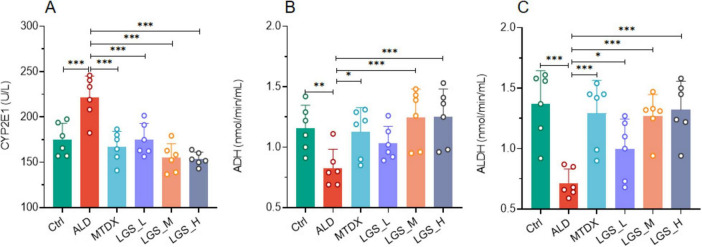
LGS enhances alcohol metabolism in a murine model of ALD. **(A)** The influence of LGS on CYP2E1 activity in ALD mice. **(B)** The influence of LGS on ADH activity in ALD mice. **(C)** The influence of LGS on ALDH activity in ALD mice (*n* = 6, *P < 0.05, **P < 0.01, ***P < 0.001). One-way ANOVA followed by Tukey test was employed to evaluate differences among multiple groups.

The liver, as the primary metabolic organ, relies on the synergistic action of various enzymes. During the process of alcohol metabolism, three key enzymes (CYP2E1, ADH and ALDH) affect the rate of alcohol metabolism. In this study, we measured the activity of those enzymes related to alcohol metabolism to assess the impact of LGS on alcohol metabolism in Figure 4.

We detected the serum levels of CYP2E1 and observed that its activity was significant increased in ALD (Figure 4A) (*p* < 0.05), which was decreased by both MTDX and LGS (*p* < 0.05). This finding suggests that LGS may alleviate oxidative stress-induced damage caused by alcohol by inhibiting the activity of CYP2E1. Furthermore, In the ALD group, the activities of ADH (Figure 4B) and ALDH (Figure 4C) decreased significantly compared to the Ctrl group, and the intervention of the two drugs in the experiment could reverse this decline (*p* < 0.05) and this effect showed an increasing trend with the increase of LGS dosage. It was worth noted that for CYP2E1 and ALDH, the low-dose group also showed significant therapeutic effects, and LGS had better regulatory effects on the three enzymes above than MTDX, although there was no statistical difference. These results indicated that LGS may reduce the toxicity of alcohol to the liver by optimizing the enzyme catalysis in the metabolic process of alcohol.

In summary, our findings supported the potential application in promoting alcohol metabolism.

### 3.2 LGS maintains the intestinal epithelial barrier in ALD mice

**FIGURE 5:**
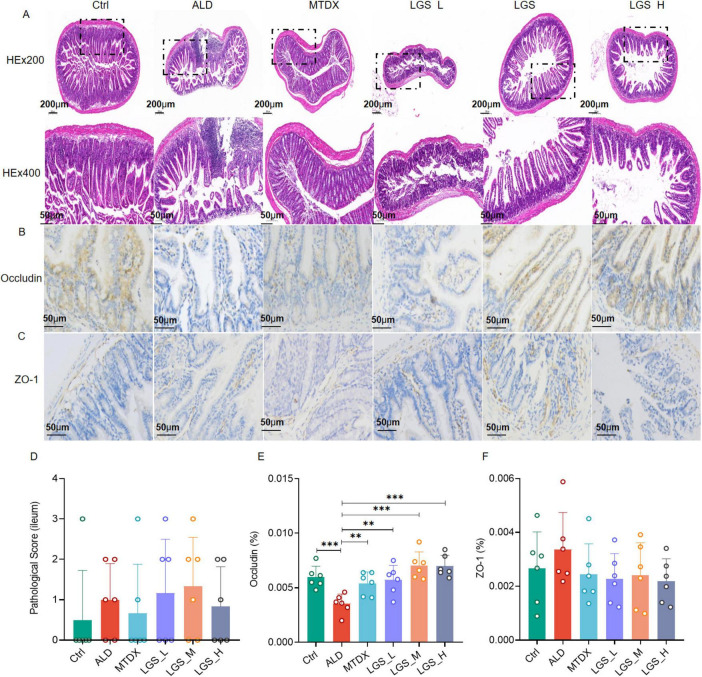
LGS ameliorates the intestinal epithelial barrier in ALD mice. **(A)** H&E staining of the ileum in mice at original magnifications of 100× and 400×. **(B)** Immunohistochemical staining for Occludin in the intestine at an original magnification of 400×. **(C)** Immunohistochemical staining for ZO-1 in the intestine at an original magnification of 400×. **(D)** Histopathological scoring of H&E staining in intestinal tissue. **(E)** Percentage of positive area expression for Occludin immunohistochemistry. **(F)** Percentage of positive area expression for ZO-1 immunohistochemistry (*n* = 6, **P < 0.01, ***P < 0.001). One-way ANOVA followed by Tukey test was employed to evaluate differences among multiple groups.

The integrity of the intestinal barrier is crucial for preventing harmful substances and pathogens from entering the bloodstream. An analysis of the intestinal mucosal structure was conducted on mice from different groups using H&E and IHC staining, as depicted in Figure 5.

In the observation of intestinal tissue (H&E staining) (Figure 5A), the intestinal tissue structure of the Ctrl remained intact, including the mucosal layer, submucosal layer, muscular layer, and serosa, with no significant pathological changes observed. The intestinal mucosal layer and submucosal layer structures of the ALD group mice exhibited structural disruption, with localized involvement of the muscularis. In the mice of LGS_M and LGS_H, the intestinal tissue structure remained intact, with no significant pathological changes observed. The intestinal mucosal layer of the LGS_L group mice was damaged, and the crypt structure of the lamina propria showed disappearance or reduction. Further analysis of the expression of occludin (Figure 5B) and ZO-1 (Figure 5C) in the ileal tissue was performed, with the brown-yellow areas representing the positive expression areas. In Ctrl group, Occludin and ZO-1 distributed in the mucosa of the small intestine evenly, with a small amount in the submucosa. Compared with the Ctrl group, the brown-yellow area in the ALD group was reduced significantly, mainly in the mucosa. After LGS intervention, the area of Occludin positivity was increased and evenly distributed, while the changes of ZO-1 in each group were not obvious. These results indicated that the mucosal structure was damaged and the integrity of the mucosal barrier was affected in ALD mice. After LGS intervention, the expression of occludin at the top of the intestinal epithelium was increased. Although the H&E pathological scoring did not show a significant advantage of LGS in improving the intestinal barrier (Figure 5D), LGS significantly enhanced the expression of occludin in the ileal tissue of ALD mice (Figure 5E). During ALD, the expression level of ZO-1 in the intestine was not changed (Figure 5F).

These results suggest that the structural integrity of the intestine in ALD mice is damaged, and LGS may repair the intestinal epithelial barrier by upregulating the expression of the intestinal channel protein occludin.

### 3.3 LGS modulates gut microbiota structure in ALD mice

**FIGURE 6:**
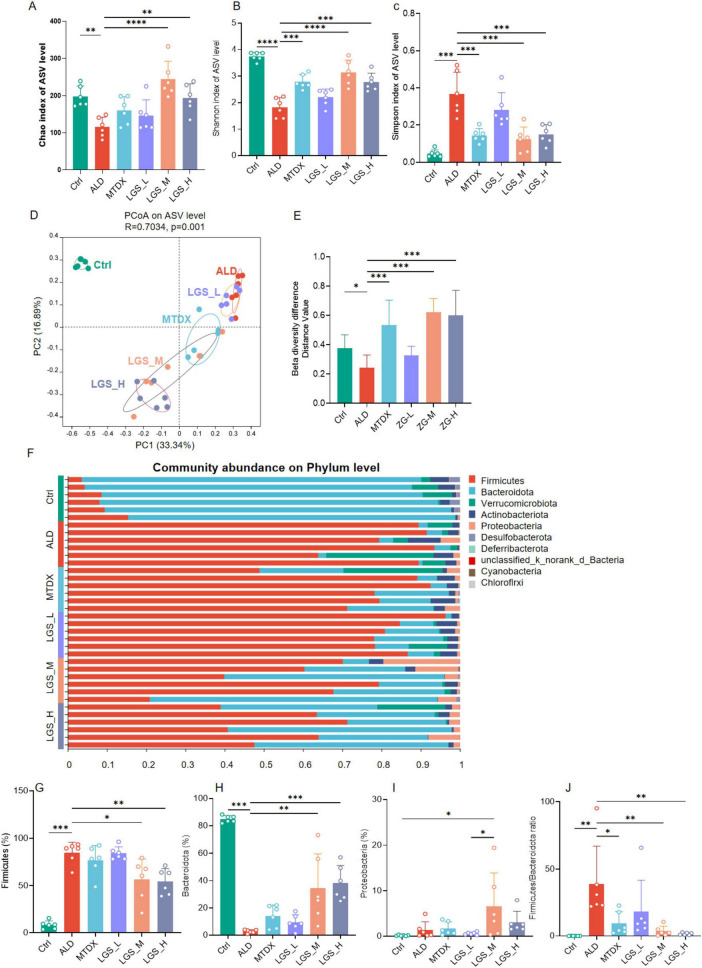
LGS improves the diversity of the gut microbiota in ALD mice and reduces the ratio of Firmicutes to Bacteroidetes. **(A)** Chao index. **(B)** Shannon index. **(C)** Simpson index. **(D)** Principal coordinates analysis (PCoA). **(E)** Differences in β diversity among groups. **(F)** Composition at the phylum level across groups (top ten abundance). **(G)** Differences of Firmicutes among groups. **(H)** Differences of Bacteroidetes among groups. **(I)** Differences of Proteobacteria among groups. **(J)** Differences of F/B (the ratio of Firmicutes to Bacteroidetes) among groups (*n* = 6, *P < 0.05, **P < 0.01, ***P < 0.001, ****P < 0.0001). One-way ANOVA followed by Tukey test was employed to evaluate differences among multiple groups.

In order to investigate the impact of LGS on the gut microbiota of ALD mice, on the 21^st^ day of the experiment, fecal samples were collected from mice in each group and subjected to 16S rRNA sequencing to observe the composition of fecal microorganisms. It should be noted that the 16S sequencing depth can be represented by the coverage index in the Alpha diversity index (Supplementary Figure 1) or provided by the average sequencing data volume of the samples (Supplementary Table 1). The coverage for each sample is above 99.9%, indicating that the sequencing depth is sufficient for subsequent analysis. The analyzed results are presented in Figure 6.

The indices of α diversity, usually refer to the Chao index, the Shannon index, and the Simpson index, are paramount in quantifying the species richness and evenness within a population across various samples. β diversity, conversely, is an analytical approach grounded in the visual interpretation of distance matrices between samples, serving to assess the variances among groups. The closer the species composition, the shorter the distance, which is graphically represented in the PCoA plot, thereby revealing the clustering phenomena among different groups. Within the scope of this study, the curves of the Chao index, the Shannon, and the Simpson indices demonstrated saturation as the quantity of random sequencing increased (Supplementary Figure 2), the α diversity in the ALD group was significantly reduced when juxtaposed with the control group (*p* < 0.05). Comparatively, this reduction was reversed following intervention with LGS. The effects of the medium and high dosage groups were more pronounced than that of the low dosage group, with the medium dosage group exhibiting a more robust recovery (Figures 6A–C). The control group and the alcohol intervention group were distinctly separated in the PCoA plot (Figure 6D), indicating the differences in the gut microbiota structure among the groups (Figure 6E). These findings suggested a decrease in the gut microbiota diversity in ALD mice, and that LGS can, to a certain extent, restore the diversity of their gut microbiota.

This study conducted an in-depth analysis of the phylum-level microbial composition of the murine gut microbiota. The findings revealed that the gut microbiota of mice was predominantly composed of the phyla Firmicutes, Bacteroidetes, Verrucomicrobia, Actinobacteria, and Proteobacteria, with Firmicutes and Bacteroidetes occupying a dominant position (Figure 6F), which is consistent with previous research findings (1). At the phylum level, in comparison to Ctrl, the ALD exhibited an enrichment of Firmicutes (Figure 6G) (*p* < 0.05) and a relative reduction in Bacteroidetes (Figure 6H) (*p* < 0.05), resulting in an elevated F/B ratio (*p* < 0.05). Furthermore, Verrucomicrobia and Actinobacteria displayed an increasing trend, while the phylum Desulfurococcales showed a decreasing trend, although these differences were not statistically significant. These findings corroborate some current studies (17–19), yet contradict others (20, 21). Following the intervention with LGS and MTDX, a significant reduction in Firmicutes was observed in LGS_M and LGS_H groups, with a re-enrichment of Bacteroidetes across all groups, particularly in LGS_M and LGS_H groups (*p* < 0.05). Proteobacteria showed an enrichment (Figure 6I) (*p* < 0.05), and the F/B ratio was reduced (Figure 6J) (*p* < 0.05). The results of this study indicated that the dysbiosis in the gut microbiota of ALD mice primarily occurred in the phyla of Bacteroidetes and Firmicutes.

Further analysis revealed that within the Bacteroidetes (Supplementary Figure 3A), the genus *Muribaculaceae* and *Alloprevotella* were predominant, while within the *Firmicutes* (Supplementary Figure 3B), the genera *Faecalibaculum, Dubosiella, Monoglobus*, and *Lactobacillus* were the main constituents. The Ctrl group was characterized by the predominance of the genus *Muribaculaceae*, whereas the ALD group was dominated by the *Faecalibaculum*, which also maintained a considerable proportion in the alcohol-fed groups. Notably, the genus *Lactobacillus* was enriched in the Ctrl group but showed a decrease following alcohol intervention, suggested that it may be beneficial for ALD (22). Additionally, in the MTDX group, the genus *Dubosiella* was enriched. Those findings suggested that the gut microbiota of ALD mice was disordered, characterized by an increased abundance of Firmicutes, represented by *Faecalibaculum*, and a significant reduction of Bacteroidetes, represented by the *Muribaculaceae*. Moreover, LGS intervention ameliorated the gut microbiota dysbiosis in ALD mice.

**FIGURE 7:**
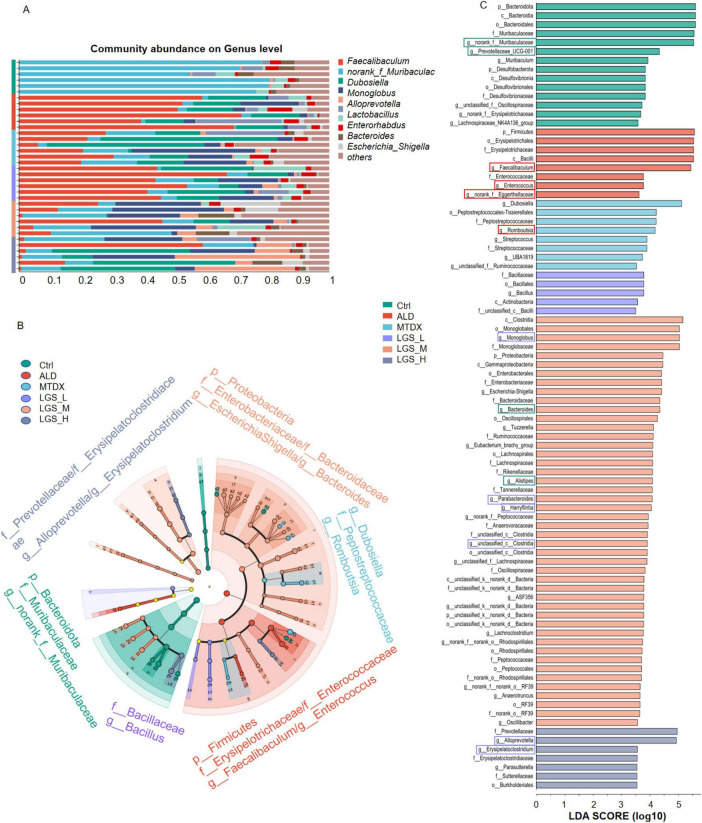
LGS improves the composition of the gut microbiota at the Genus Level in ALD mice. **(A)** Composition of the Intestinal Microbiota at the Genus Level among groups. **(B)** Multilevel Species Hierarchical Tree Diagram Linear Discriminant Analysis Effect Size (LDA = 3.5, *n* = 6). **(C)** LEfSe Analysis among groups. The green box indicates a decrease in ALD and an increase after LGS intervention, the red box indicates an enrichment in ALD and a decrease after LGS intervention, and the purple box indicates an increase only in the LGS group.

To further investigate the impact of LGS on the microbial structure of mice in each group, the composition of the gut microbiota at the genus level, and LEfSe Analysis (LDA = 3.5, *n* = 6) were employed to compare the microbial structures among the groups in Figure 7.

The top 10 genera in abundance are shown in Figure 7A. Utilizing LEfSe analysis (LDA = 3.5, *n* = 6), we compared the microbial structures of different groups of mice, noticed potential biomarkers among groups (Figures 7B, C). In Ctrl, biomarkers mainly included *g_norank_f_Muribaculaceae.* Besides *g_Prevotellaceae_UCG-001*, *g_Lachnospiraceae_NK4A136_group* were abundant in the Ctrl group and may play a positive role in maintaining the balance of the gut microbiota and host health. Conversely, the *g_Faecalibacterium*, and *g_Enterococcus* were identified in the ALD group, which may be associated with the development of ALD. In the intervention groups, we found that *g_Dubosiella*, and *g_Romboutsia* in MTDX group, while the LGS_L was characterized by *g_Bacillus*. The biomarkers for the LGS_M included *g_Escherichia-Shigella*, and *g_Bacteroides*. The biomarkers for the LGS_H were *g_Alloprevotella*, and *g_Erysipelatoclostridium.*

**FIGURE 8:**
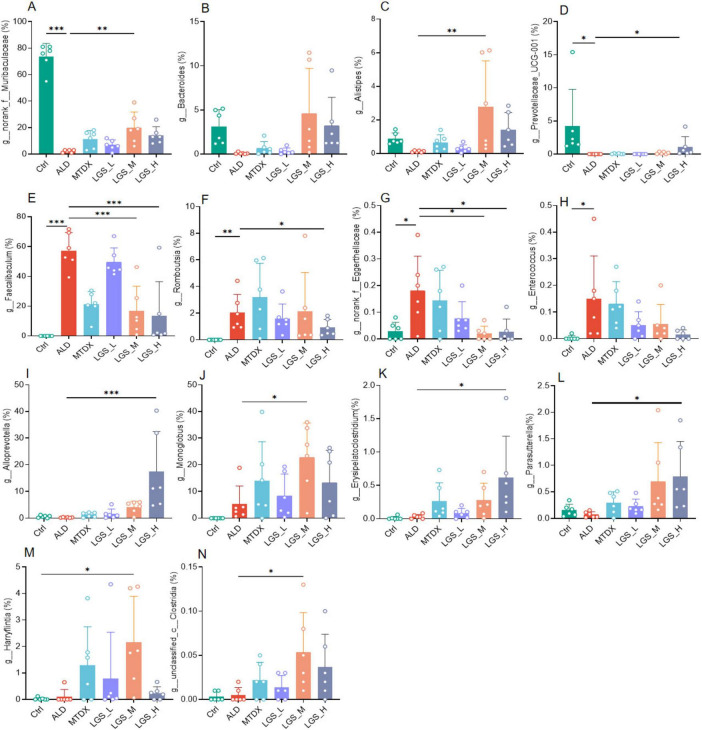
LGS intervention specifically alters gut microbes in ALD mice. **(A)** Differences of *g_norank_f_Muribaculaceae* among groups. **(B)** Differences of *g_Bacteroides* among groups. **(C)** Differences of *g_Alistipes* among groups. **(D)** Differences of *g_Prevotellaceae_UCG-001* among groups. **(E)** Differences of *g_Faecalibaculum* among groups. **(F)** Differences of *g_Romboutsia* among groups. **(G)** Differences of *g_norank_f_Eggerthellaceae* among groups. **(H)** Differences of *g_Enterococcus* among groups. **(I)** Differences of *g_Alloprevotella* among groups. **(J)** Differences of *g_Monoglobus* among groups. **(K)** Differences of *g_Erysipelatoclostridium* among groups. **(L)** Differences of *g_Parasutterella* among groups. **(M)** Differences of *g_Harryflintia* among groups. **(N)** Differences of *g_unclassified_c_Clostridia* among groups (*n* = 6, *P < 0.05, **P < 0.01, ***P < 0.001). One-way ANOVA followed by Tukey test was employed to evaluate differences among multiple groups.

A further comparison and analysis of the contents of various bacterial genera among the groups showed that LGS may against ALD by suppressing or enriching specific bacterial genera in Figure 8. We found some unique microbial genera, appeared only in specific groups, may play an important role in the microbial composition or have specific functions in Supplementary Table 2.

Initially, the *g_norank_f_Muribaculaceae* (Figure 8A) (*p* < 0.05), *g_Bacteroides* (Figure 8B), *g_Alistipes* (Figure 8C), *g_Prevotellaceae_UCG-001* (Figure 8D) (*p* < 0.05) decreased in the ALD compared with Ctrl group, which may be related to the imbalance of the gut microbiota in ALD mice. However, after LGS intervention, the abundance of these genera rebounded, indicated that LGS may restore the balance of the gut microbiota targeting these genera. Furthermore, the *g_Faecalibaculum* (Figure 8E), *g_Romboutsia* (Figure 8F), *g_norank_f_Eggerthellaceae* (Figure 8G), and *g_Enterococcus* (Figure 8H) were significantly enriched in the ALD group, and may be related to the pathogenesis of ALD. After LGS intervention, the abundance of these genera was reversed, indicating that LGS may alleviate the pathological process of ALD by regulating their abundance. Additionally, it was also noted that some unique genera appeared or became enriched in association with LGS, such as *Alloprevotella* (Figure 8I), *Monoglobus* (Figure 8J), *Erysipelatoclostridium* (Figure 8K), *Parasutterella* (Figure 8L), *Harryflintia* (Figure 8M), and *unclassified_c_Clostridia* (Figure 8N), which only increased after LGS intervention (*p* < 0.05), and these genera might have been specifically targeted by LGS.

Overall, LGS regulated gut microbiota in mice through restoring ALD-mediated microbial changes and specifically inducing some gut microbes.

### 3.4 LGS promotes hexanoic acid production and regulates GPR43/GLP-1 pathway in ALD mice

**FIGURE 9:**
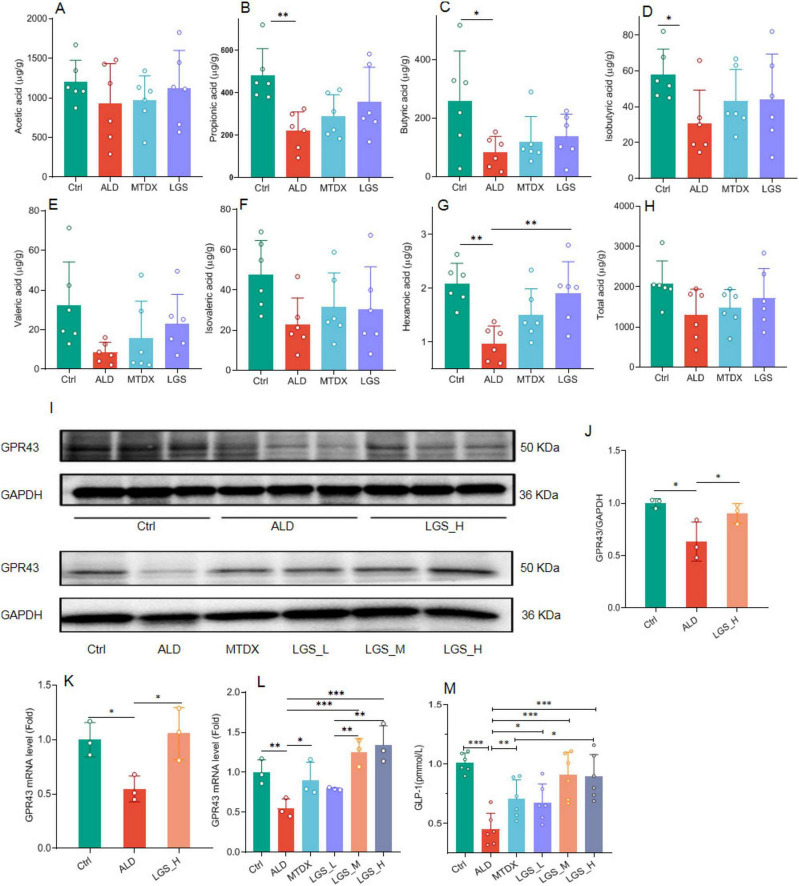
The influence of LGS on the SCFAs/GPR43/GLP-1 pathway in ALD mice. **(A)** The effect of LGS on the cecal content of acetic acid among groups. **(B)** The effect of LGS on the content of propionic acid among groups. **(C)** The effect of LGS on the content of butyric acid among groups. **(D)** The effect of LGS on the content of isobutyric acid among groups. **(E)** The effect of LGS on the content of valeric acid among groups. **(F)** The effect of LGS on the content of isovaleric acid among groups. **(G)** The effect of LGS on the content of hexanoic acid among groups. **(H)** The effect of LGS on the content of total acid among groups. **(I)** Detection of the expression of GPR43 in mouse ileal tissue by Western blot. **(J)** Semi-quantitative analysis of the expression of GPR43 in mouse ileal tissue of Western Blot. **(K,L)** Quantitative analysis of the expression of *GPR43* in mouse ileal tissue of qPCR. **(M)** The concentration of serum GLP-1 (*n* = 6, *P < 0.05, **P < 0.01, ***P < 0.001). One-way ANOVA followed by Tukey test was employed to evaluate differences among multiple groups.

SCFAs are the metabolic products of the gut microbiota and are closely related to ALD. In this study, the content and composition of SCFAs in the cecal contents of mice were determined by GC-MS in Figure 9.

In the study of SCFAs (Figures 9A–H), it was observed that the levels of various SCFAs in the cecum of mice in the ALD group were decreased. The administration of LGS restored the content of SCFAs in the cecal contents. Further analysis revealed that the levels of propionic acid (Figure 9B), butyric acid (Figure 9C), isobutyric acid (Figure 9D), and hexanoic acid (Figure 9G) were significantly reduced opposed to the Ctrl group (*p* < 0.05). After treatment, there was a certain degree of recovery in the levels of the aforementioned SCFAs, with a statistically significant improvement observed in the recovery of hexanoic acid (*p* < 0.05). The propionic acid content was also remarkably increased in LGS group, however, no statistical difference was found. The results suggested that LGS increased the total acid content in the intestinal tissue of ALD mice and restored the levels of various SCFAs, especially the hexanoic acid.

GLP-1 is an important target of SCFAs, and SCFAs can stimulate the release of GLP-1 from intestinal epithelial cells through the GPR43 pathway (23, 24), to improve liver fat deposition (25, 26). Western blot analysis in the ileal tissue showed a significant reduction in the expression of intestinal GPR43 in ALD mice (Figure 9J) (*p* < 0.05), which reversed by LGS (*p* < 0.05), which was confirmed again in PCR detection (Figures 9K, L). Concurrently, the detection of serum GLP-1 content showed changes consistent with GPR43 (Figure 9M), which was significantly reduced in the ALD group and could be increased by MTDX and LGS. However, the extent to which MTDX increased was similar to that of the low-dose LGS. The medium and high doses of LGS were able to restore the GLP-1 content to a level comparable to that of the Ctrl group, and the LGS_H group was superior to the MTDX group. Therefore, LGS may exert an anti-ALD effect through the hexanoic acid/GPR43/GLP-1 pathway.

## 4 Discussion

**FIGURE 10:**
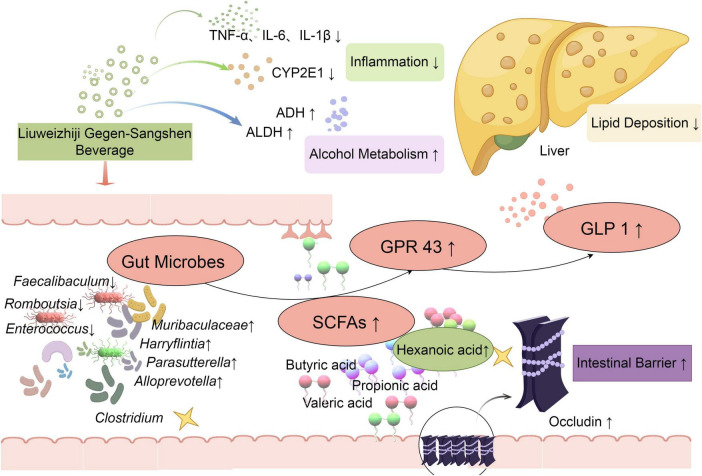
LGS protects against ALD in mice through the gut microbiota mediated SCFAs/GPR43/GLP-1 pathway. Levels of ALT, AST, TNF-^α^, IL-6, and IL-1β were decreased by LGS to mitigate alcoholic liver injury in mice. The activity of ethanol metabolism enzymes, ALDH and ADH, was enhanced, which improved the capacity for alcohol metabolism. The function of maintaining intestinal barrier was well preserved by LGS through reducing epithelial damage and increasing the expression of Occludin. Moreover, the structure of the gut microbiota in mice with alcoholic liver disease was significantly modulated by LGS, which restored alcohol-induced microbial alterations, specifically mediated the enrichment (*Muribaculaceae*, *Alloprevotella, Parasutterella, Harryflintia*), the suppression (*Faecalibaculum, Romboutsia, Enterococcus*), or the production (*Clostridium*). Further research indicated that the production of SCFA (hexanoic acid) in the cecum was increased by LGS, which promoted the increase of ethanol-mediated ileal GRP43 expression and increased the levels of serum GLP-1 to reduction of lipid deposition in the liver.

In this study, the anti-ALD effect of LGS and its possible mechanism was investigated by the ALD murine model mice treated with LGS. Our results highlight that LGS exerts a remarkable protective effect on ALD mice through the gut microbiota mediated hexanoic acid/GPR43/GLP-1 pathway (Figure 10).

The pathogenesis of ALD is complex, and has yet been fully understood (27). Chronic alcohol consumption can lead to the development of ALD, potentially driven by metabolic and immunologic factors induced by alcohol and its metabolites, including reactive oxygen species (ROS) and proinflammatory cytokines (28). Specifically, alcohol exposure boosts the activity of the hepatic CYP2E1 enzyme, which increases ROS production, causing hepatic mitochondrial dysfunction, and stimulates *de novo* lipogenesis (28). Additionally, damage- and pathogen-associated molecular patterns activate specific receptors in non-parenchymal liver cells, such as Kupffer cells, hepatic stellate cells (HSCs), and lymphocytes, causing hepatocyte death and the infiltration of proinflammatory cells like neutrophils and macrophages into the liver (29). Various forms of hepatocyte death, including apoptosis, necroptosis, pyroptosis, and ferroptosis, have been reported to coexist in ALD (29). In severe cases, such as cirrhosis and severe alcohol-associated hepatitis, there is a significant hepatocyte degeneration. Liver sinusoidal endothelial cell dysfunction also contributes to ALD development. Furthermore, alcohol promotes global protein acetylation, disrupting clathrin-mediated endocytosis, a process that affects the uptake of macromolecules and the trafficking of receptor-ligand complexes, leading to metabolic imbalance (30).

Organ crosstalk has also emerged as a significant issue in ALD. Chronic alcohol intake disrupts the gut microbiome and its barrier function, allowing endotoxin leakage into the portal circulation and facilitating the transfer of triglycerides from adipose tissue to the liver through lipolysis (28). Notably, alterations in gut microbiota play a crucial role in the pathogenesis of ALD, as alcohol-induced changes in gut microbe compromise the intestinal epithelial barrier and trigger proinflammatory mediators.

Currently, no effective drugs for the treatment of ALD have been approved, highlighting the urgency of finding new therapeutic approaches (28). Some of drugs and therapies have been evaluated in clinical trials (Table 1), mainly targeting hepatocyte death and regeneration, inflammation and gut microbiome. In particular, fecal microbiota transplantation as well as some of probiotics have shown promising results in alleviating ALD in human. Therefore, targeting the gut microbiota presents new prospect in ALD treatment.

**TABLE 1:** Ongoing trails for ALD and the targets.

Candidate drug	Targets	Status	References
IL-22 agonist (F-562)	Targeting hepatocyte death and regeneration	Ongoing phase IIb trials	(31)
IL-1R inhibitor	Antiinflammation	Ongoing phase II trial	(32)
Anti-LPS (hyperimmune bovine colostrum enriched with IgG)	Antiinflammation	Ongoing phase IIa clinical trial	NCT01968382
ASK-1 inhibitor (selonsertib GS-4997)	Targeting apoptosis	No benefits from a phase IIa trial	NCT02854631
Metadoxine	Anti-ROS	Short-term survival benefit	(33)
Microbiome and gut-liver axis	Healthy donor fecal microbiota transplantation (FMT)	A randomized clinical trial revealed survival benefit at 90 days in patients with severe alcoholic hepatitis	(34)
Microbiome and gut-liver axis	*Lactobacillus casei*	Improve lipid metabolism and regulate intestinal flora disorders in patients with alcoholic liver injury	(35)
Microbiome and gut-liver axis	*Lactobacillus subtilis*/*Streptococcus faecium*	Restoration of bowel flora and improvement of LPS in patients with alcoholic hepatitis	(36)
Microbiome and gut-liver axis, anti-ROS, improving alcohol metabolism	LGS	Improve lipid metabolism and inflammation	(7–10) and Current research

The present study focused on a Chinese medicine-derived herbal beverage, LGS. LGS, composed of six herbal components that are both edible and medicinal, was not observed to exhibit clear toxic side effects in previous clinical applications, offering a novel therapeutic choice for ALD. Previous studies (7–10) have demonstrated that LGS protected against ALD rats through alleviation of lipid metabolism and relief of inflammation in liver via regulating several signaling pathways. In LO-2 cells, LGS was found to inhibit ROS through regulating CYP2E1 and enhance alcohol metabolism through increasing ADH1 and ALDH2 expression (11). However, whether LGS had an impact on gut microbiota remains unexplored. Our previous report demonstrated that LGS mainly contained polyphenols, flavonoids and polysaccharides (12). Notably, the LGS polysaccharides have shown specific modulation *in vitro* on certain gut microbes that were potentially beneficial to health. Therefore, it is of our primary interests to investigate the impact of LGS on the gut microbiota and gut-liver axis.

As AFL and ASH were identified as the most prevalent clinical phase among patients with excessive alcohol consumption, and as the initial stage of ALD, recognized for its theoretical reversibility in treatment, the murine ALD model was established by the NIAAA method in our study to fit this stage of the disease. And we confirmed that in ALD mice, alcohol intake led to hepatic steatosis, elevated liver function tests, and serum inflammatory mediators. After the intervention of LGS, the aforementioned indicators were reversed, which confirmed the effectiveness of LGS for ALD via attenuating liver steatosis and injury (decreased AST, ALT, TC, TG and HDL-C, increased LDL-C, and alleviated histopathological scores in liver), relieving inflammation (decreased TNF-α, IL-6, and IL-1β) and enhancing alcohol metabolism (increased ADH and ALDH, and reduced CYP2E1 activity). These findings were consistent with previous reports based on rat ALD models.

Importantly, we newly found in the present study that LGS exerted a profound role in regulating the gut microbiota and the gut-liver axis. LGS effectively maintained the function of the intestinal barrier by reducing epithelial damage and increasing the expression of Occludin. In the analysis of the gut microbiota, it was observed that the F/B ratio in ALD mice significantly increased, which is consistent with the biological markers of obesity (37). The intervention of LGS not only reversed this change but also specifically mediated the enrichment of several bacterial genera, which have been proven to be associated with intestinal inflammation and lipid metabolism, potentially becoming the potential targets of LGS action. In particular, supplementation with LGS specifically mediated enrichment of several bacterial genera (*Alloprevotella, Monoglobus, Erysipelatoclostridium, Parasutterella, Harryflintia* and *unclassified_c_Clostridia*).

In the intestinal tract of mammals, the phyla Firmicutes and Bacteroidetes are identified as the two predominant bacterial phyla, the ratio of which, known as the F/B ratio, serves as an important indicator for assessing the balance of the gut ecosystem (1). Further analysis revealed that *Faecalibacterium* within the phylum Firmicutes and the *Bacteroidaceae* within the phylum Bacteroidetes are key microbial groups influencing the gut ecology. *Muribaculaceae* were found to be significant. Recent research has confirmed that both of them are known to produce SCFAs, and *Muribaculaceae* is negatively correlated with liver function abnormalities (38).

The *Alistipes* genus, which is negatively correlated with the occurrence of liver fibrosis and colitis (39). *Prevotellaceae_UCG-001* has been shown to improve colitis induced by *Clostridium* difficile (40), and its enrichment in the gut is associated with the significant improvement of abnormal liver lipid metabolism related to Type 2 Diabetes Mellitus by Jerusalem artichoke (41). *Alloprevotella*, which may play an important role in improvements of liver functions and reduction of colitis susceptibility (42). This suggested that they may alleviate pathological changes in ALD by regulating liver lipid metabolism and producing anti-inflammatory substances. In our experiments, these microbial communities were reduced in ALD, and notably increased following intervention with LGS, which corroborated their beneficial effects on alcoholic liver disease.

Regarding some of the genera that significantly enriched in ALD, they were notably suppressed following intervention with LGS or MTDX. The *Romboutsia*, a Gram-positive bacterium, has been identified as non-alcoholic steatohepatitis marker in the progression of non-alcoholic fatty liver disease (NAFLD) (43). Given the similarities between NAFLD and ALD in terms of liver fat accumulation and inflammation, *Romboutsia* may play a similar role in ALD. The *Eggerthellaceae* genus is positively correlated with the total gastrointestinal symptoms (including constipation) in children with autism spectrum disorder (44) and negatively correlated with the levels of Claudin 3 in patients with cirrhosis (45). Cirrhosis is one of the severe complications of ALD, indicating that *Eggerthellaceae* may affect the progression of ALD by influencing the intestinal barrier function and liver inflammatory response. Furthermore, the *Enterococcus* genus may improve dextran sulfate sodium-induced colitis by increasing acetate production, reducing butyrate production, and regulating the expression of GPR43 (46). Overall, these genera may affect the progression of ALD by influencing the balance of gut microbiota, liver metabolism, and inflammatory responses through various mechanisms.

We have also noted some genera enriched in the LGS group, such as *Monoglobus* and *Erysipelatoclostridium*. *Monoglobus* is a pectin-degrading bacteria. LGS contained rich pectic polysaccharides, which may lead to enrichment of the pectin-degrading *Monoglobus*. It is reported that the *Erysipelatoclostridium* genus is associated with obesity (47) and is related to a lower risk of intrahepatic cholestasis of pregnancy (ICP) (48). The *Erysipelatoclostridium* plays a protective role in cirrhosis and primary biliary cholangitis (PBC) (49). Similarly, *Parasutterella, Harryflintia* and *Clostridia*, have been found to be negatively associated with ALD, sugar and fat metabolism, or intestinal inflammation (50–55). In terms of quantity, those genera only predominated after the LGS intervention.

Gut microbiota plays an important role in host health through generation of metabolites (56). The main microbial products include fermentation products (SCFAs, branched SCFAs), bile acid metabolites (secondary bile acids), amino acids degradants (indole), bacterial proteins and products (LPS, Amuc 1100), bioactive lipids (12-hydroxyeicosatetraenoic acid), and exosomes (57). These molecules can act on various host cell receptors, such as toll-like receptors (TLRs), peroxisome proliferator-activated receptor alpha (PPARα), aryl hydrocarbon receptor (AhR), G-protein-coupled receptors (e.g., GPR41/43/119 and TGR5), and endocannabinoid receptors, to regulate host signaling pathways and impact physiological functions such as the intestinal barrier, immune function, insulin resistance and host metabolism (57). Under conditions of gut barrier disruption, the bacteria can translocate into liver via portal vein and trigger host immune response.

The SCFAs, primarily comprising acetic acid, propionic acid, and butyric acid, have been identified as the principal end products of gut microbial fermentation. Indeed, they have been demonstrated to confer benefits not only to intestinal health but also to hepatic protection through modulation of energy metabolism, inflammatory responses, and lipid metabolism (58–60). However, the SCFAs were typically found in lower abundance in ALD, which was consistent with our experimental findings (61). After LGS intervention, the content of SCFAs increases, which is related to the enrichment of bacteria producing SCFAs in the gut microbiota, such as *Muribaculaceae, Alloprevotella, Parasutterella* and *Harryflintia* (62–65). In addition, *Romboutsia* was found to be potentially associated with the decrease in SCFAs, and it was inhibited after LGS intervention (66). The production of SCFAs required dietary fiber as a substrate, but *Enterococcus* population was reduced due to the decrease in dietary fiber (67). It is noteworthy that after the intervention of LGS, the levels of various SCFAs did not significantly rebound as we had expected, which may be related to the primary production by the *Firmicutes*, a phylum that enriched in the context of ALD. The improvement of hexanoic acid was not negligible, indicated that although valeric acid and hexanoic acid are present in low quantities among SCFAs, they are still worth further exploration and may play a pivotal role in certain aspects.

Valeric acid and hexanoic acid, strictly speaking, are medium-chain fatty acids, yet they appear in studies related to SCFAs. Valeric acid, is closely related to the body’s energy metabolism as a direct form of energy storage. It has been demonstrated to ameliorate the impaired glucose homeostasis and insulin sensitivity in type 2 diabetes patients (68), showing a negative correlation with HDL-C (69). Hexanoic acid was produced by *Clostridium* (70, 71), which mainly utilized ethanol and glucose as substrates and grew anaerobically with ethanol and acetic acid as the only energy sources (72). Additionally, *norank_f_Muribaculaceae* (73), *Eubacterium*, *Ruminococcaceae*, and *Lachnospiraceae* (74) involved in the production of hexanoic acid (75). As the only saturated fatty acid that increases blood sugar levels (76), hexanoic acid has been confirmed that the inhibition of α-amylase activity is achieved, which has a clear effect on lowering postprandial blood glucose levels (77). On the one hand, it can promote appetite and weight gain; on the other hand, as a medium-chain fatty acid, it has a tendency to promote balanced metabolism, maintain optimal insulin sensitivity, and even promote the basal and insulin-dependent phosphorylation of the Akt-mTOR pathway (78). The deficiency of hexanoic acid was found to be associated with neurological disorders such as cognitive impairment (79–81). In studies related to SCFAs, some research suggests that low levels of hexanoic acid are associated with intestinal damage (74, 82). Oppositely, another study found that medium-chain fatty acids, represented by hexanoic acid, promote intestinal inflammatory responses, while SCFAs, represented by butyric acid, restore the intestinal barrier in pregnant women with diabetes (83).

GPR43 has been identified as one of the downstream receptors of SCFAs (23, 84, 85), and can be activated by hexanoic acid directly (86). Then activation of GPR43 is coupled with the downstream Gq/11 and Gi/G0 signaling pathway (87). The secretion of GLP-1 is triggered (23), which promotes glucose uptake and thereby improves glucose homeostasis within the body (24). The GPR43/GLP-1 axis has been recognized for its role in promoting insulin secretion and inhibiting glucagon secretion (88). Some studies also demonstrated that GPR43 specifically activated by SCFAs in the M2 macrophages facilitated the maintenance of gut barrier function (89). For the liver, GLP-1 has been found to inhibit the deposition of liver fat (90), and further research has confirmed that its antagonists may be beneficial for the treatment of NAFLD (91). In addition, GLP1/GLP1R were found to affect cell apoptosis (92), oxidative stress (93), inflammatory responses (94, 95), and even autophagy (96) and membrane transport (97) through multiple pathways, suggesting that LGS might influence the aforementioned mechanisms by modulating GLP1/GLP1R. It is showed that pre-treatment with liraglutide (a GLP-1 analog) significantly inhibited the M1 polarization of macrophages during liver ischemia-reperfusion injury (95). Combined with our experimental results, LGS has been shown to increase the content of SCFA (hexanoic acid) within the intestinal tract, along with enhanced expression of GPR43. The secretion of GLP-1 was also increased. Therefore, LGS treatment was able to specifically regulate gut microbiota (increased *Muribaculaceae, Alloprevotella, Parasutterella* and *Harryflintia*) to generate SCFA (hexanoic acid) which activated GPR43. The activated GPR43 is believed to maintain gut barrier function and improve glucose homeostasis through GLP-1.

In summary, LGS exerts a significant protective effect on ALD mice through the gut microbiota-mediated production of hexanoic acid and activating GPR43/GLP-1 pathway, which might contribute to the maintenance of host glucose homeostasis and gut barrier function. Taken together with previous reports, LGS might be an effective treatment for ALD through multiple targets involving anti-inflammation, modulation of lipid and alcohol metabolism and gut microbiota, which may be attributable to its varied constituents of polyphenols, flavonoids and polysaccharides.

## 4.1 Research prospects and limitations

The model employed in this study was found to be more suitable for the early stages of AFL and AH in ALD. Although several targets within the gut-liver axis had been identified in the study, such as the gut microbiota (*Muribaculaceae, Alloprevotella, Parasutterella and Harryflintia*), SCFAs (*hexanoic acid*), and GPR43/GLP-1, the specific mechanisms, especially those delving into the cellular level, still required further research for validation. For instance, fecal transplantation can be performed to validate the role of gut microbiota regulation. GPR43 knockout mice can be used for validation of its role. The affected microbial strains can be further confirmed for ALD protection. In addition, the exploration of constituents of LGS would benefit for understanding the active fractions.

## 5 Conclusion

In summary, the findings of this study lead us to conclude that LGS has a definite protective effect on ALD mice. Consistent with previous reports, LGS alleviated liver steatosis, attenuated liver injury and inflammation and enhanced alcohol metabolism. It was newly found that LGS remarkably alleviated ALD-associated gut barrier function abnormality and gut dysbiosis. The attenuated gut dysbiosis was characterized as: (1) recovering the ALD-mediated alteration of microbial structure; (2) enriching some of unique microbial strains (*Alloprevotella, Monoglobus*, *Erysipelatoclostridium*, *Parasutterella*, *Harryflintia*, and *unclassified_c_Clostridia*). Moreover, Following LGS intervention, the microbial SCFA metabolite, hexanoic acid, was significantly increased, which was associated with activation of intestinal GPR43 and production of GLP-1. Therefore, the modulation of the gut microbiota and activation of the SCFAs/GPR43/GLP-1 signaling pathway may contribute to the anti-ALD effect of LGS.

Although this study has certain limitations, these discoveries provide a new perspective (particularly the modulation of gut microbiota) for further exploration of the application of LGS in the treatment of ALD. Future research should focus on the specific components of LGS, the affected microbial genera, and the types of SCFAs to gain a deeper understanding of the mechanisms of LGS. In addition, fecal transplantation or GPR43 inhibition should be performed to confirm their roles in ALD alleviation by LGS. These studies would provide a scientific basis for the development of new therapeutic strategies.

## Data Availability

The raw data supporting the conclusions of this article will be made available by the authors, without undue reservation.

## Footnotes

1
https://bio-cloud.aptbiotech.com/

2
https://cloud.majorbio.com

3
http://huttenhower.sph.harvard.edu/LEfSe
