# Supplementary material for: Liuweizhiji Gegen-Sangshen beverage protects against alcoholic liver disease in mice through the gut microbiota mediated SCFAs/GPR43/GLP-1 pathway
Source: Front Nutr. 2024 Dec 13;11:1495695. doi: 10.3389/fnut.2024.1495695 (PMC11673767; doi:10.3389/fnut.2024.1495695)
Supplement: Supplementary file 1 [file Data_Sheet_1.docx]

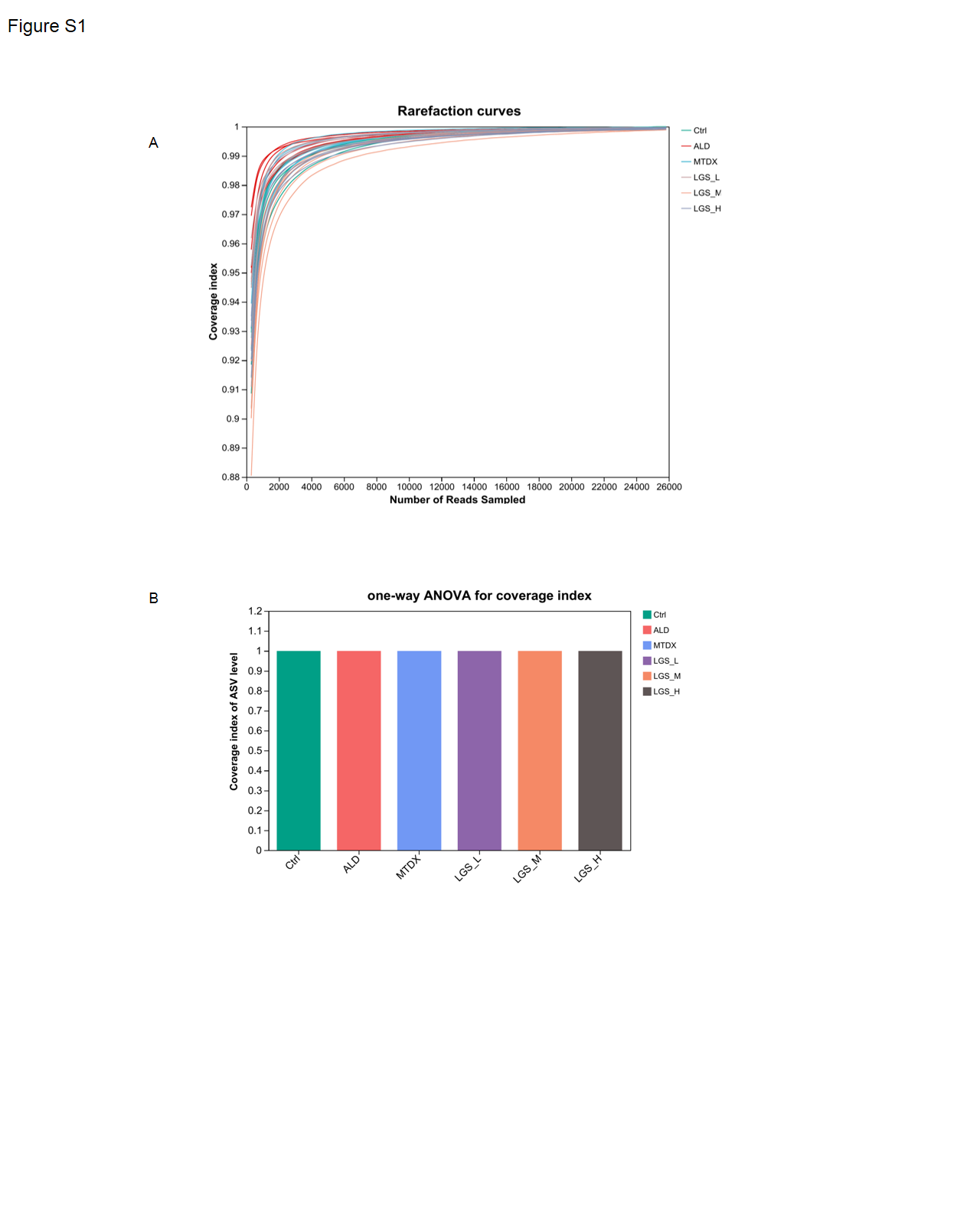


Figure S1 .α diversity rarefaction curve. (A) Coverage Index. (B) One-way ANOVA for Coverage Index


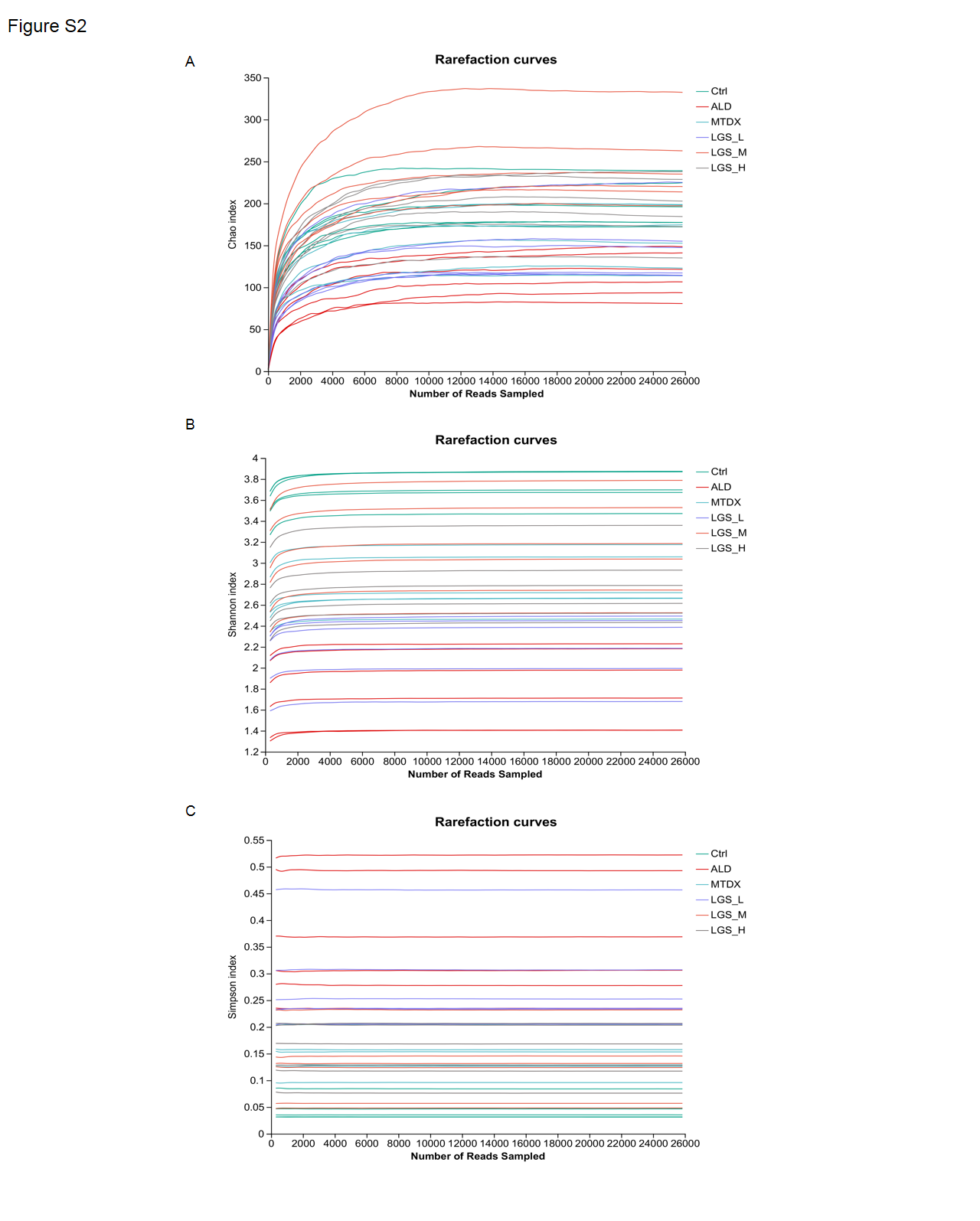


Figure S 2 α diversity rarefaction curve. (A) Chao Index. (B) Shannon Index. (C) Simpson Index.


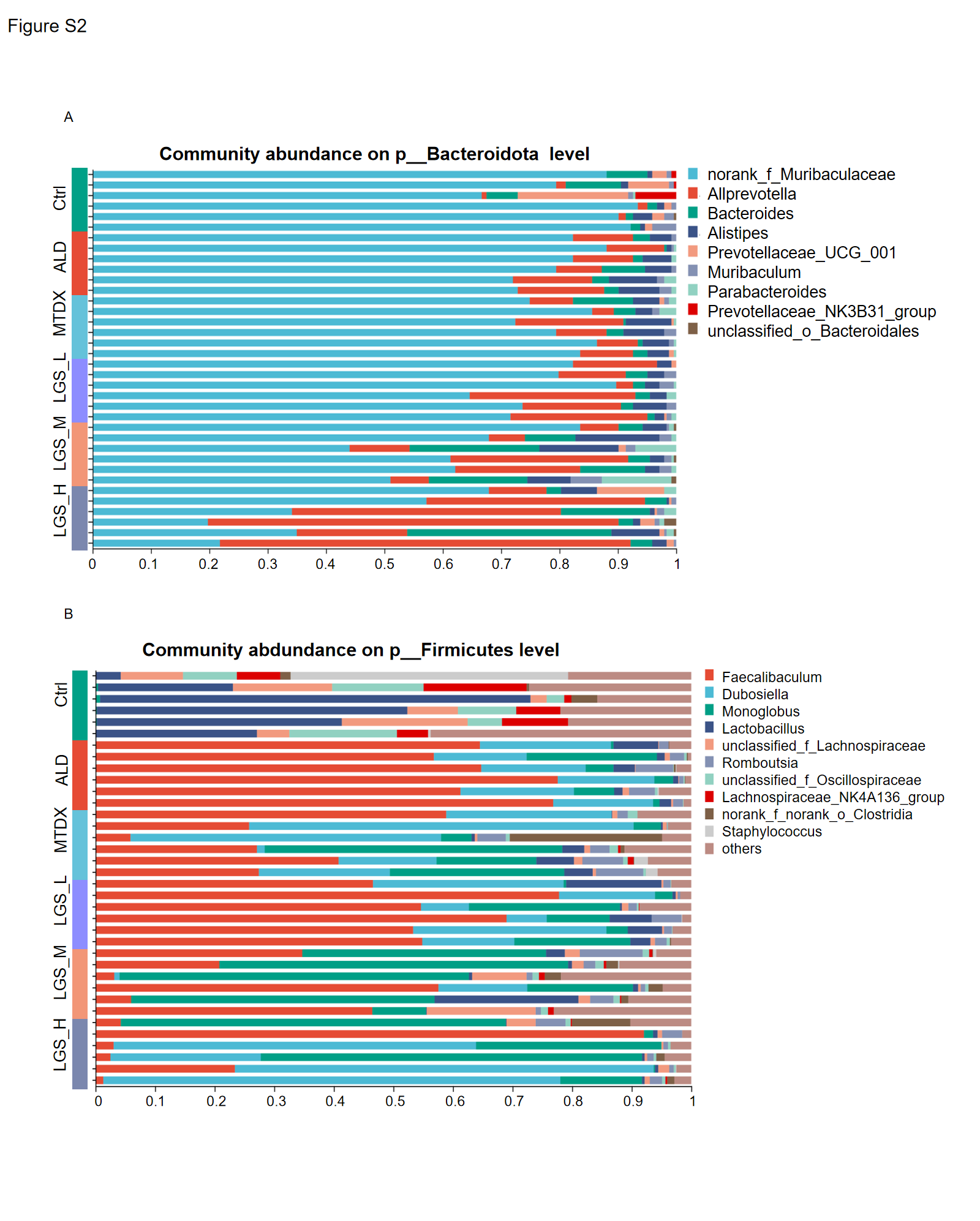
Figure S3 Composition of the Bacteroidetes phylum within groups. (B) Composition of the Firmicutes phylum within groups (top ten abundance).

Table S 1 Sequence information statistics.

| Sample_info | Seq_num | Base_num  (bp) | Mean_length (bp) | Min_length  (bp) | Max_length  (bp) |
| --- | --- | --- | --- | --- | --- |
| Ctrl_1 | 82286 | 34768070 | 422.527161 | 253 | 512 |
| Ctrl_2 | 67013 | 28279511 | 422.000373 | 255 | 509 |
| Ctrl_3 | 63168 | 26696042 | 422.619713 | 272 | 517 |
| Ctrl_4 | 63607 | 26898512 | 422.886035 | 270 | 444 |
| Ctrl_5 | 62304 | 26321899 | 422.475266 | 220 | 430 |
| Ctrl_6 | 107356 | 45352798 | 422.452383 | 252 | 526 |
| ALD_1 | 51104 | 21797820 | 426.538431 | 216 | 446 |
| ALD_2 | 59551 | 25129610 | 421.984685 | 252 | 433 |
| ALD_3 | 54830 | 23204254 | 423.203611 | 255 | 434 |
| ALD_4 | 55195 | 23565317 | 426.946589 | 283 | 433 |
| ALD_5 | 63519 | 26636925 | 419.353658 | 260 | 434 |
| ALD_6 | 59551 | 25377018 | 426.139242 | 259 | 437 |
| MTDX_1 | 65601 | 27638068 | 421.30559 | 325 | 517 |
| MTDX_2 | 79610 | 33870924 | 425.460671 | 226 | 430 |
| MTDX_3 | 52632 | 22086725 | 419.644418 | 228 | 436 |
| MTDX_4 | 53833 | 22347374 | 415.124069 | 257 | 465 |
| MTDX_5 | 56551 | 23738687 | 419.77484 | 255 | 439 |
| MTDX_6 | 55642 | 23353632 | 419.712304 | 277 | 432 |
| LGS_L_1 | 58793 | 25142988 | 427.652748 | 270 | 437 |
| LGS_L_2 | 54753 | 23329508 | 426.086388 | 259 | 437 |
| LGS_L_3 | 67686 | 28466783 | 420.571211 | 259 | 443 |
| LGS_L_4 | 63092 | 26760533 | 424.15097 | 258 | 432 |
| LGS_L_5 | 56874 | 24148975 | 424.604828 | 258 | 432 |
| LGS_L_6 | 59618 | 25169981 | 422.187611 | 277 | 515 |
| LGS_M_1 | 57870 | 24133788 | 417.034526 | 262 | 430 |
| LGS_M_2 | 60924 | 25373939 | 416.485113 | 252 | 436 |
| LGS_M_3 | 66710 | 27862370 | 417.664068 | 220 | 431 |
| LGS_M_4 | 63120 | 26660643 | 422.380276 | 258 | 432 |
| LGS_M_5 | 57418 | 23892099 | 416.108172 | 277 | 434 |
| LGS_M_6 | 67780 | 28647250 | 422.650487 | 272 | 515 |
| LGS_H_1 | 68001 | 28255180 | 415.511242 | 237 | 467 |
| LGS_H_2 | 53704 | 22859855 | 425.663917 | 266 | 450 |
| LGS_H_3 | 59532 | 25099447 | 421.612696 | 277 | 443 |
| LGS_H_4 | 47728 | 20036628 | 419.808666 | 325 | 432 |
| LGS_H_5 | 58501 | 24960955 | 426.675698 | 284 | 526 |
| LGS_H_6 | 53269 | 22594032 | 424.149731 | 284 | 432 |

**Table S 2** Unique genera of bacteria in each group.

| **Group** | **Number** | **Description** | **Current Research** |
| --- | --- | --- | --- |
| Ctrl | 15 | *g_Prevotellaceae_NK3B31_group (*86.67%), *g_norank_f_norank_o_SJA-15,g_Ruminococcus, g_norank_f_cvE6, g_norank_f_norank_o_norank_c_Clostridia,g_Candidatus_Arthromitus, g_Candidatus_Stoquefichus, g_unclassified_f_Rhodocyclaceae, g_norank_f_norank_o_C10-SB1A, g_Butyricicoccus,g_Microbacterium,g_norank_f_A4b, g_Eubacterium_siraeum_group, g_Comamonas, g_IMCC26207* | The low abundance of *g_Prevotellaceae_NK3B31_group* can serve as a biomarker for fatty liver disease, which may be related to the increased degradation of lysine and histidine, and its increased abundance may be associated with the improvement of alcoholic liver disease ([1](#_ENREF_1), [2](#_ENREF_2)). |
| ALD | 5 | *g_Aerococcus (40%), g_Clostridioides, g_unclassified_f_Rhizobiaceae, g_unclassified_f_Eggerthellaceae, g_unclassified_o_Lactobacillales* | The genus *g_Aerococcus* has been identified as an opportunistic pathogen in relation to type 2 diabetes mellitus ([3](#_ENREF_3)). The role of this microorganism in the study of alcoholic liver disease is controversial, with some studies suggesting a potentially beneficial effect and others suggesting a detrimental effect ([4](#_ENREF_4), [5](#_ENREF_5)). |
| MTDX | 7 | *g_Clostridium_sensu_stricto_1 (56.14%), g_GCA-900066575 (16.20%), g_Peptococcus (9.66%), g_Defluviitaleaceae_UCG-011, g_norank_f_norank_o_Oscillospirales, g_Paludicola, g_Clostridium_innocuum_group.* | The *g_Clostridium_sensu_stricto_1* produces short-chain fatty acids (acetic acid, butyric acid) and promotes the intestinal mucosal barrier ([6](#_ENREF_6)).  The increase in the abundance of *g_GCA-900066575* is related to the inhibition of liver fat deposition by propionic acid and may also be related to the increase in the levels of acetic, propionic and butyric acids ([7](#_ENREF_7)). And it also supplements the enrichment-related improvements of Akkermansia muciniphila in liver damage and gallic acid in liver fat deposition ([8](#_ENREF_8)).  The *g_Peptococcus* is a protective factor for ALD translates to *g_Peptococcus* serves as a protective factor for ALD ([9](#_ENREF_9)). |
| LGS_L | 4 | *g_Acinetobacter (58.33%), g_unclassified_o_Peptostreptococcales-Tissierellales, g_Jeotgalicoccus, g_Acidovorax.* | *g_Acinetobacter* is currently dedicated to the study of antibiotic resistance and water purification technologies, and the optimization of liver lipid metabolism may be associated with the generation of lipase ([10](#_ENREF_10)). |
| LGS_M | 11 | *g_norank_f_Eubacterium_coprostanoligenes_group (78.19%), g_Anaerovorax, g_unclassified_p_Proteobacteria, g_Sphingomonas, g_unclassified_o_Enterobacterales, g_Acetatifactor, g_Flavonifractor, g_UCG-004, g_Erysipelotrichaceae_UCG-003, g_Family_XIII_UCG-001, g_unclassified_f_Rikenellaceae.* | The *g_norank_f_Eubacterium_coprostanoligenes_group* is a type of microorganism capable of producing SCFAs. It is not only associated with the sheen of hair ([11](#_ENREF_11)), but also has the potential to alleviate drug-induced liver damage ([12](#_ENREF_12)).  The butyrate-producing *g_Flavonifractor* can alleviate intestinal inflammation ([13](#_ENREF_13)). |
| LGS_H | 5 | *g_norank_f_norank_o_Bacteroidales (33.33%), g_unclassified_o_Lachnospirales, g_Ruminococcus_torques_group, g_Xanthomonas, g_unclassified_c_Gammaproteobacteria (16.67%).* | The *g_Ruminococcus*_torques group is positively correlated with the abundance of short-chain fatty acids, a potential beneficial bacterium for intestinal anti-inflammation, and a protective factor for alcoholic liver disease ([9](#_ENREF_9)). |

Note: “g_” represents “genus", "s_" represents "species", "f_" represents "family", "o_" represents "order" and "c_" represents "class".

**Reference**

1. Driuchina A, Hintikka J, Lehtonen M, Keski-Rahkonen P, O'Connell T, Juvonen R, et al. Identification of Gut Microbial Lysine and Histidine Degradation and CYP-Dependent Metabolites as Biomarkers of Fatty Liver Disease. MBIO. 2023;14(1):e0266322.<https://doi.org/10.1128/mbio.02663-22>

2. Yi Z, Liu X, Liang L, Wang G, Xiong Z, Zhang H, et al. Antrodin A from Antrodia camphorata modulates the gut microbiome and liver metabolome in mice exposed to acute alcohol intake. FOOD FUNCT. 2021;12(7):2925-37.<https://doi.org/10.1039/d0fo03345f>

3. Ye J, Meng Q, Jin K, Luo Y, Yue T. Phage cocktail alleviated type 2 diabetes by reshaping gut microbiota and decreasing proinflammatory cytokines. Applied microbiology and biotechnology. 2024;108(1):9.<https://doi.org/10.1007/s00253-023-12912-7>

4. Lv XC, Wu Q, Cao YJ, Lin YC, Guo WL, Rao PF, et al. Ganoderic acid A from Ganoderma lucidum protects against alcoholic liver injury through ameliorating the lipid metabolism and modulating the intestinal microbial composition. FOOD FUNCT. 2022;13(10):5820-37.<https://doi.org/10.1039/d1fo03219d>

5. Zheng M, Pi X, Li H, Cheng S, Su Y, Zhang Y, et al. Ganoderma spp. polysaccharides are potential prebiotics: a review. Critical reviews in food science and nutrition. 2024;64(4):909-27.<https://doi.org/10.1080/10408398.2022.2110035>

6. Wang L, Zhang Y, Liu L, Huang F, Dong B. Effects of Three-Layer Encapsulated Tea Tree Oil on Growth Performance, Antioxidant Capacity, and Intestinal Microbiota of Weaned Pigs. Frontiers in veterinary science. 2021;8:789225.<https://doi.org/10.3389/fvets.2021.789225>

7. Guo J, Wang P, Cui Y, Hu X, Chen F, Ma C. Protective Effects of Hydroxyphenyl Propionic Acids on Lipid Metabolism and Gut Microbiota in Mice Fed a High-Fat Diet. NUTRIENTS. 2023;15(4).<https://doi.org/10.3390/nu15041043>

8. Zhou C, Zhang W, Lin H, Zhang L, Wu F, Wang Y, et al. Effect of theaflavin-3,3'-digallate on leptin-deficient induced nonalcoholic fatty liver disease might be related to lipid metabolism regulated by the Fads1/PPARδ/Fabp4 axis and gut microbiota. Frontiers in pharmacology. 2022;13:925264.<https://doi.org/10.3389/fphar.2022.925264>

9. Zhang L, Zi L, Kuang T, Wang K, Qiu Z, Wu Z, et al. Investigating causal associations among gut microbiota, metabolites, and liver diseases: a Mendelian randomization study. Front Endocrinol (Lausanne). 2023;14:1159148.<https://doi.org/10.3389/fendo.2023.1159148>

10. Lu Z, Yao C, Tan B, Dong X, Yang Q, Liu H, et al. Effects of Lysophospholipid Supplementation in Feed with Low Protein or Lipid on Growth Performance, Lipid Metabolism, and Intestinal Flora of Largemouth Bass (Micropterus salmoides). Aquaculture nutrition. 2022;2022:4347466.<https://doi.org/10.1155/2022/4347466>

11. Wu D, Zhao P, Wang C, Huasai S, Chen H, Chen A. Differences in the intestinal microbiota and association of host metabolism with hair coat status in cattle. Frontiers in microbiology. 2024;15:1296602.<https://doi.org/10.3389/fmicb.2024.1296602>

12. Wang L, Dong XL, Qin XM, Li ZY. Investigating the inter-individual variability of Astragali Radix against cisplatin-induced liver injury via 16S rRNA gene sequencing and LC/MS-based metabolomics. Phytomedicine : international journal of phytotherapy and phytopharmacology. 2022;101:154107.<https://doi.org/10.1016/j.phymed.2022.154107>

13. Haskey N, Estaki M, Ye J, Shim RK, Singh S, Dieleman LA, et al. A Mediterranean Diet Pattern Improves Intestinal Inflammation Concomitant with Reshaping of the Bacteriome in Ulcerative Colitis: A Randomised Controlled Trial. Journal of Crohn's & colitis. 2023;17(10):1569-78.<https://doi.org/10.1093/ecco-jcc/jjad073>
